# Association of Iba1-Positive Macrophages and B7-H3-Positive Tumor Cells with Tumor Growth Kinetics in WHO Grade II Meningioma: A Pilot Watch-and-Wait Cohort Study

**DOI:** 10.3390/cancers18101545

**Published:** 2026-05-10

**Authors:** Eiji Ito, Masasuke Ohno, Mao Yokota, Shunichiro Kuramitsu, Toru Nagasaka, Toshiaki Inomo, Tadashi Watanabe, Mitsugu Fujita

**Affiliations:** 1 Department of Neurosurgery, Aichi Medical University, 1-1 Yazakokarimata, Nagakute 480-1195, Japan; itou.eiji.160@mail.aichi-med-u.ac.jp (E.I.); yokota.mao.107@mail.aichi-med-u.ac.jp (M.Y.); watanabe.tadashi.598@mail.aichi-med-u.ac.jp (T.W.); 2 Department of Neurosurgery, Aichi Cancer Center, 1-1 Shikanodono, Chikusa-ku, Nagoya 464-8681, Japan; m.ono@aichi-cc.jp; 3 Department of Neurosurgery, Nagoya Medical Center, 4-1-1 Sannomaru, Naka-ku, Nagoya 460-0001, Japan; skramitz@yahoo.co.jp; 4 Association of Medical Artificial Intelligence Curation, Sakae Members Office Building, Room 505, 4-16-8 Sakae, Naka-ku, Nagoya 460-0008, Japan; toru-ngy@umin.ac.jp; 5 Department of Neurosurgery, Tsushima City Hospital, 3-73 Tachibana-cho, Tsushima 496-0038, Japan; inomo.toshiaki.495@mail.aichi-med-u.ac.jp; 6 Center for Medical Education and Clinical Training, Kindai University Faculty of Medicine, 1-14-1 Miharadai, Minami-ku, Sakai 590-0197, Japan

**Keywords:** WHO grade II meningioma, tumor immune microenvironment, tumor-associated macrophages, B7-H3, tumor growth kinetics, immunophenotype, prognosis

## Abstract

Background/Objectives: WHO grade II meningiomas show highly variable growth rates that are difficult to predict using conventional histology. We investigated relationships between immune components within the tumor immune microenvironment (TIME) and explored their association with tumor growth kinetics. Methods: We retrospectively evaluated a small, preselected cohort of 15 patients with WHO grade II meningiomas who underwent initial “watch-and-wait” observation with serial preoperative MRI. Notably, this cohort represents a specific clinical subset—tumors deemed initially observable—and may not be representative of all WHO grade II meningiomas. Densities of Iba1-positive macrophages (TAMs) and B7-H3-positive tumor cells were quantified by digital pathology. The relative growth rate (RGR) was calculated from serial MRI. Results: A strong inverse correlation was observed between TAM density and B7-H3-positive tumor cell density (Spearman’s *R* = −0.921, *p* < 0.001). Individual immune components did not show significant linear correlations with RGR; on median-split stratification, high B7-H3-positive tumor cell density was associated with a nominally significant difference in RGR (*p* = 0.0428, unadjusted). Low TAM density showed a non-significant trend in the same direction. Conclusions: Our findings generate the hypothesis that WHO grade II meningiomas exhibit an inverse relationship between TAMs and B7-H3 and that macrophage-poor/B7-H3-high and macrophage-rich/B7-H3-low patterns may differ in preoperative growth behavior. These observations are derived from a small, preselected cohort of tumors managed with an initial “watch-and-wait” strategy and require validation in broader populations.

## Simple Summary

Predicting the growth rate of WHO grade II meningiomas remains a clinical challenge. We analyzed 15 cases using the relative growth rate (RGR), a metric that quantifies intrinsic tumor expansion from serial imaging. We observed an inverse correlation between the density of tumor-associated macrophages (TAMs; Iba1-positive) and B7-H3-positive tumor cells. Exploratory analyses suggested that macrophage-poor/B7-H3-high tumors tended to show higher preoperative growth rates than macrophage-rich/B7-H3-low tumors. Because of the small, preselected cohort, these observations should be interpreted as hypothesis-generating.

## 1. Introduction

The World Health Organization (WHO) classifies grade II meningiomas as intermediate malignancies based on histology. However, these tumors exhibit heterogeneous clinical behavior that is not fully explained by conventional histological grading [1,2]: some progress or recur rapidly while others remain dormant for years [3,4]. Recent studies have identified prognostic groups (MG1–MG4) that extend beyond histological grading [5]. Specifically, the MG1 subtype is characterized by high immune infiltration and favorable outcomes (“immunogenic”), whereas the MG4 subtype exhibits high proliferation rates, chromosomal instability, and poor prognosis (“proliferative”). However, comprehensive molecular profiling is not routinely available in many centers [6]. Consequently, there is a pressing need to identify accessible biomarkers, such as those found in the tumor immune microenvironment (TIME), that reflect these intrinsic tumor behaviors and can serve as practical tools for risk stratification.

Within this framework, the balance between immune surveillance and immune evasion in the TIME is a plausible determinant of clinical heterogeneity [7,8]. This balance reflects the origin of meningiomas in immunologically active meninges [9,10]. We focused on tumor-associated macrophages (TAMs), the dominant immune infiltrate in meningiomas, and on the B7-H3 immune checkpoint for its dual role in immune evasion and tumor proliferation [7,11,12]. Concurrent evaluation of TAM density and B7-H3 expression may thus serve as a surrogate for the host defense–tumor escape equilibrium underlying clinical variability in meningiomas.

We used the relative growth rate (RGR) from serial imaging, which normalizes growth to baseline volume [13,14,15,16]. Therefore, this exploratory pilot study primarily aimed to characterize the relationship between the densities of Iba1-positive macrophages (defined as TAMs) and B7-H3-positive tumor cells and, secondarily, to explore the association of these immune components with tumor growth kinetics measured by RGR. We hypothesized that, reflecting the known biological heterogeneity, these immune components might segregate into distinct immunophenotypic profiles associated with differences in RGR.

## 2. Materials and Methods

### 2.1. Study Design and Ethical Approval

This single-center retrospective cohort study was conducted in accordance with the principles of the Declaration of Helsinki. Ethical approval was obtained from the Aichi Medical University Hospital Ethics Committee (approval number: 2024-246; approval date: 4 February 2025). Given that all data were collected as part of routine clinical care, individual informed consent was waived in accordance with the Ethical Guidelines for Medical and Health Research Involving Human Subjects in Japan. Instead, an opt-out notice detailing the study objectives and ensuring the right to refuse participation was made available on the hospital website. This procedure was explicitly approved by the Institutional Review Board. No patients opted out of the study. All personal identifiers were removed before analysis.

### 2.2. Patient Selection

This study extended our previous TIME investigation in meningiomas [17] by assembling a grade II subset with serial preoperative imaging. The clinical and radiological records of consecutive patients who underwent surgical resection for WHO grade II intracranial meningiomas at Aichi Medical University Hospital between January 2014 and December 2023 were reviewed. The specific inclusion criteria were as follows: (1) histopathologically confirmed WHO grade II meningioma; (2) availability of suitable contrast-enhanced T1-weighted magnetic resonance imaging (MRI) data from at least two time points before treatment to calculate tumor growth (necessitating a preoperative “watch-and-wait” imaging interval); and (3) sufficient tissue samples for immunohistochemical analysis. Patients with neurofibromatosis type 2, other tumor-predisposition syndromes, spinal or extracranial tumors, or multifocal disease were excluded.

### 2.3. Volumetric Analysis and Calculation of Growth Metrics

Contrast-enhanced T1-weighted MRI DICOM datasets from two time points were imported into an image-processing workstation (Zaio; Ziosoft, Tokyo, Japan). All examinations used thin-slice protocols with a slice thickness of ≤1.5 mm. An experienced neurosurgeon, blinded to the clinical outcomes, manually outlined tumor borders on every axial slice using a freehand tool for each scan pair. Volumes were computed by slice summation:

*V* [cm^3^] = *∑A_i_* × (*t* + *g*) (1)

where *A_i* is the lesion area on slice *i*, *t* is slice thickness, and *g* is the inter-slice gap. To evaluate tumor growth dynamics, two mathematical models were assessed. First, the monthly volume change (*MVC*, cm^3^/month) was calculated as a linear measure of absolute volume expansion. Second, the relative growth rate (*RGR*, %/month) was calculated under an exponential model, normalizing growth to the baseline tumor volume. The exponential model was used as the established standard for meningioma volumetry; comparison with alternative models (e.g., Gompertzian) was not feasible given *n* = 15.

Δ*t_months_* = *Δt_days_*/30.44 (2)

Relative growth rate (*RGR*, %/month) was calculated as

*RGR* = [(*V*_2_/*V*_1_)^1/Δ*tmonths*^ − 1] × 100 (3)

Monthly volume change (*MVC*, cm^3^/month) was calculated as

*MVC* = (*V*_2_ − *V*_1_)/Δt_*months*_ (4)

where *V*_1_ and *V*_2_ represent the tumor volumes at the initial and follow-up imaging time points, respectively. To assess reproducibility, tumor volumes were measured twice by a single observer (EI) under the identical protocol, and the intraclass correlation coefficient (ICC) and mean absolute percentage difference were calculated. The mean volume value was used for the final analysis.

### 2.4. Histological Processing and Immunohistochemistry

This procedure was conducted in accordance with previous studies [17,18,19]. Briefly, formalin-fixed, paraffin-embedded meningioma tissue was sectioned at 4 µm and mounted on silane-coated slides. One section was stained with hematoxylin and eosin for morphological assessment. Adjacent sections underwent multicolor immunohistochemistry using the following primary monoclonal antibodies (mAbs): anti-Iba1 (clone EPR16589; Abcam, Cambridge, UK), anti-CD4 (clone EPR6855; Abcam, Cambridge, UK), and anti-B7-H3/CD276 (clone EPR20115; Abcam, Cambridge, UK). The Histofine Simple Stain system (Nichirei, Tokyo, Japan) was used for detection. Distinct chromogenic substrates were applied to each target according to the manufacturer’s instructions. The substrates were diaminobenzidine (DAB; Nichirei Tokyo, Japan) for CD4 (brown), HistoGreen (Cosmo Bio, Tokyo, Japan) for B7-H3 (light green), and First Red II (Nichirei) for Iba1 (red). Areas of overlap between the Iba1 and B7-H3 signals appeared dark brown in the composite staining. All sections were counterstained with hematoxylin. To ensure staining consistency, all slides were stained in a single batch using a standardized protocol with consistent incubation times and reagent lots.

### 2.5. Image Analysis

Whole-slide images were obtained at 20× magnification using a NanoZoomer-SQ system (Hamamatsu Photonics, Hamamatsu, Japan) and imported into QuPath software (version 0.6.0) for analysis [20]. To minimize technical variance, all samples were stained in a single batch, and whole-slide digital scanning was performed on the same day with identical settings. Tumor regions were delineated using automated pixel classification followed by manual verification. For cellular classification, a supervised object classifier (Random Trees algorithm; OpenCV implementation within QuPath v0.6.0) was trained via an iterative, specimen-by-specimen active learning approach: annotation regions for each cell class were drawn progressively across all 18 specimens, with the classifier retrained after each addition. Performance stabilized after approximately seven iterations; the final classifier (version 18), incorporating all 18 specimens, was applied uniformly for quantification. Given the exploratory nature of this pilot study, this classifier served as a semi-automated tool for objective, uniform counting rather than a standalone algorithm with formal performance metrics (e.g., sensitivity and specificity); a held-out validation set was not feasible, and accuracy was verified by an expert neuro-oncologist who visually inspected detection results across every specimen. The classifier distinguished ten cell classes: Tumor− (B7-H3-negative tumor cells), Tumor+ (weak B7-H3-positive), Tumor++ (strong B7-H3-positive), Iba1-positive macrophage (TAMs), Iba1-negative macrophage, Iba1-negative monocyte, CD4-positive T cells, debris, endothelial cells, and RBC. For downstream analysis, Tumor+ and Tumor++ were merged as B7-H3-positive tumor cells. TAM density was defined as the count of Iba1-positive macrophage cells only; Iba1-negative macrophage and monocyte cells were excluded as they lacked the defining Iba1 chromogenic signal. Cell densities were expressed as the number of positive cells per square millimeter of tumor area.

### 2.6. Statistical Analysis

Statistical analyses were performed in EZR version 1.68 (Saitama Medical Center, Jichi Medical University, Saitama, Japan) [21,22], a graphical interface for R version 4.5.0. No statistical sample size calculation was performed a priori, as the sample size was determined by the number of available cases during the study period; therefore, this study was designed as an exploratory pilot analysis. To assess the precision of the estimates and post hoc statistical power for Spearman’s rank correlation, 95% confidence intervals and power values were calculated using Fisher’s z-transformation. Continuous variables are presented as medians with ranges. The normality of continuous variables was assessed using the Shapiro–Wilk test. Given the non-normal distributions, non-parametric methods were used without data transformation. Specifically, associations between continuous variables were evaluated using Spearman’s rank correlation coefficients, while group comparisons were performed using the Mann–Whitney U test. To evaluate the stability and robustness of the correlation between key immune components, a leave-one-out cross-validation (LOOCV) approach was implemented. For visualization and exploratory purposes, patients were dichotomized into “low” and “high” density groups based on the median value of each cellular subset. Statistical significance was defined as a two-sided *p* < 0.05.

## 3. Results

### 3.1. Clinical Characteristics of the Patient Cohort

The clinical and radiological records were initially reviewed for 18 consecutive patients. Three patients were excluded due to incomplete imaging data, and the remaining 15 patients who underwent resection for WHO grade II meningiomas were included in the final analysis. Their clinical characteristics are summarized (Table 1). The extent of resection was assessed using the Simpson grading system. Gross total resection (Simpson Grade I–II) was achieved in 7 cases (46.7%), while 8 cases underwent subtotal resection (Simpson Grade III–IV). Postoperative radiation therapy was administered in 8 patients. The median imaging interval for the serial preoperative MRI was 63.0 days (range, 33–1815 days). The intraclass correlation coefficient for the repeated volumetric measurements was >0.95, and the mean absolute percentage difference between duplicate measurements was 2.57%. The median RGR, used as a proxy for proliferative activity, was 4.5%/month. The median MVC, reflecting absolute volume increase, was 1.51 cm^3^/month. The median postoperative follow-up duration was 36 months (range, 11–136 months). No cases of recurrence were identified within this cohort during this period. This may be attributed to the relatively short follow-up duration (median 36 months) and the high rate of postoperative adjuvant therapy (53.3%), in addition to the selection of cases with an initial dormant course.

We also analyzed the impact of tumor location on the immune microenvironment and growth kinetics. Skull base tumors (*n* = 4) showed comparable TAM density (median: 797.7 cells/mm^2^) and B7-H3-positive tumor cell density (median: 3453.9 cells/mm^2^) to non-skull base tumors (*n* = 11) (Mann–Whitney U test, *p* = 0.133 and *p* = 0.103, respectively). Similarly, there was no significant difference in RGR between skull base and non-skull base tumors (*p* = 0.95). Furthermore, postoperative radiation therapy status was not associated with preoperative RGR (Mann–Whitney U test, *p* = 0.867) or immune biomarker densities (*p* = 0.336 for TAMs, *p* = 0.536 for B7-H3). Additionally, the extent of resection (Simpson Grade) was not significantly associated with preoperative RGR (Spearman’s *R* = 0.380, *p* = 0.162).

### 3.2. Immunohistochemical Characterization of the TIME

**Figure 1:**
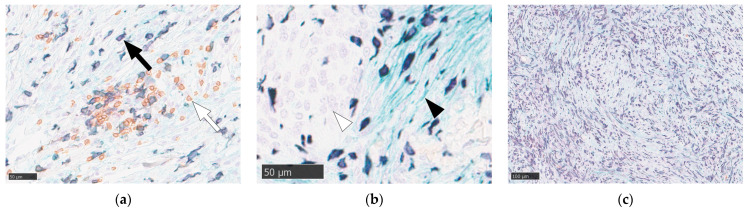
Representative images of triple immunostaining in WHO grade II meningiomas. Iba1-positive macrophages are stained red, B7-H3 expression on tumor cell membranes is light green, and CD4-positive helper T cells are brown. (**a**) Macrophages appearing dark brown due to signal overlap are indicated by a black arrow, and CD4-positive T cells are indicated by a white arrow. (**b**) High-magnification view showing heterogeneous B7-H3 expression. A B7-H3-positive tumor cell with distinct light green membrane staining (black arrowhead) is adjacent to B7-H3-negative tumor cells (white arrowhead). (**c**) Example of a tumor with dense macrophage infiltration (Case 9, 3268 cells/mm^2^, which is above the cohort median of 2053.3 cells/mm^2^). Numerous Iba1-positive macrophages (dark brown) were distributed throughout the tumor parenchyma. Scale bars: 50 µm (**a**,**b**) and 100 µm (**c**).

**Figure 2:**
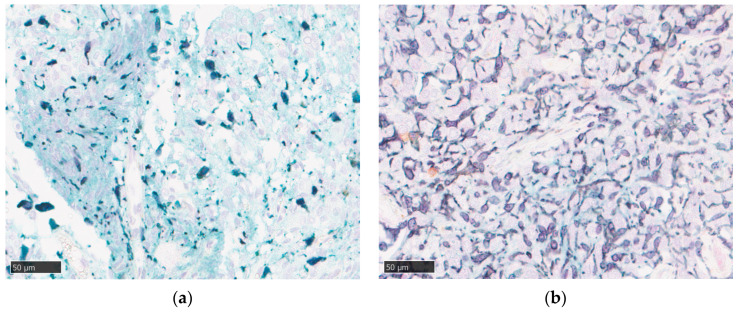
Representative immunohistochemical images of distinct immunophenotypes in WHO grade II meningiomas. (**a**) Representative case of the macrophage-poor/B7-H3-high phenotype (Case 11). This tumor exhibited a high relative growth rate (RGR) of 24.6%/month. The image reveals sparse infiltration of Iba1-positive macrophages (dark brown) accompanied by diffuse and strong B7-H3 expression (light green) on tumor cell membranes, representative of the macrophage-poor/B7-H3-high pattern observed in this cohort. (**b**) Representative case of the macrophage-rich/B7-H3-low phenotype (Case 3). This tumor showed a low preoperative growth rate (RGR = −2.9%/month). The tissue is characterized by dense infiltration of Iba1-positive macrophages (dark brown) with minimal B7-H3 expression on tumor cells, representative of the macrophage-rich/B7-H3-low pattern observed in this cohort. Nuclei were counterstained with hematoxylin. Scale bars: 50 µm.

The TIME of WHO grade II meningiomas was subsequently characterized using multicolor immunohistochemistry and digital image analysis. This analysis revealed the presence of both Iba1-positive TAMs and CD4-positive helper T cells distributed throughout the tumor parenchyma (Figure 1a). B7-H3 immunoreactivity was specifically localized to the cell membranes of tumor cells. Notably, the expression of B7-H3 in tumor cells was heterogeneous, with some neoplastic cells exhibiting distinct membrane staining and others being negative (Figure 1b). The density of immune cell infiltration varied markedly among the cases, with some tumors displaying dense macrophage accumulation (Figure 1c). Representative histological images contrasting these phenotypes are shown (Figure 2): Case 11 (low TAM [890.8 cells/mm^2^]/high B7-H3 [3432.3 cells/mm^2^], RGR = 24.6%/month) versus Case 3 (high TAM [2820.6 cells/mm^2^]/low B7-H3 [1198.1 cells/mm^2^], RGR = −2.9%/month).

### 3.3. Inverse Relationship Between TAM Infiltration and B7-H3 Expression

**Figure 3:**
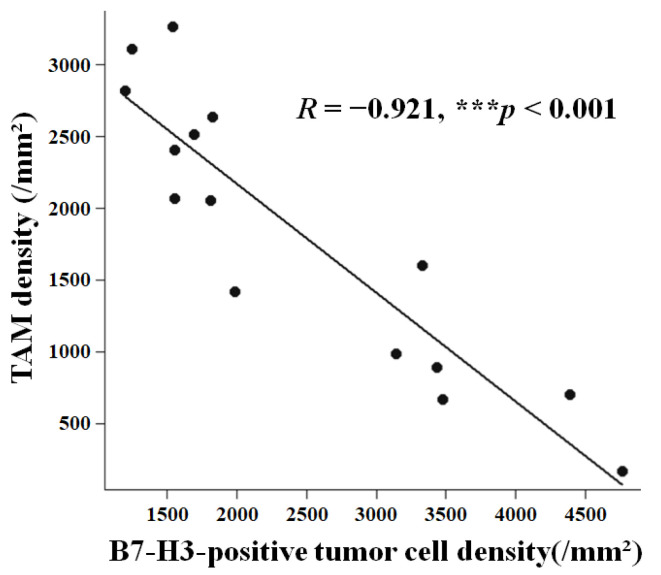
Inverse relationship between TAMs and B7-H3 in WHO grade II meningiomas. Scatter plot analysis demonstrated a strong inverse correlation between TAM density and B7-H3-positive tumor cell density across patients (Spearman’s *R* = −0.921, *p* < 0.001). ***, *p* < 0.001.

The relationship between these key cellular components within the TIME was investigated. We found a significant inverse correlation between the density of Iba1-positive TAMs and that of B7-H3-positive tumor cells (Spearman’s *R* = −0.921, 95% CI: −0.974 to −0.775, *p* < 0.001; Figure 3). In contrast, the density of CD4-positive helper T cells showed no significant correlation with either B7-H3-positive tumor cell density (Spearman’s *R* = −0.168, 95% CI: −0.626 to 0.377, *p* = 0.550) or TAM density (Spearman’s *R* = 0.096, 95% CI: −0.437 to 0.580, *p* = 0.732). To ensure that this association was not driven by extreme values, we performed a sensitivity analysis by excluding the cases with the highest TAM density (Case 9) and the highest B7-H3-positive tumor cell density (Case 7). The inverse correlation remained robust in this restricted cohort (*n* = 13, Spearman’s *R* = −0.896, 95% CI: −0.969 to −0.680, *p* < 0.001). Furthermore, to rigorously validate the stability of this key finding within our limited sample size, we performed LOOCV. Across all 15 iterations, Spearman’s rank correlation coefficient between TAM density and B7-H3-positive tumor cell density remained highly consistent (range, *R* = −0.903 to −0.947; all *p* < 0.001). This supports the robustness of the observed inverse relationship, suggesting that the association is not solely driven by any single case.

### 3.4. Exploratory Association of Immune Component Densities with Tumor Growth Rate

The quantitative relationship between these immunodistinctive cell populations and tumor growth kinetics was examined (Table 2). As the majority of variables, including tumor growth metrics and specific immune cell densities, demonstrated non-normal distributions according to the Shapiro–Wilk test, non-parametric methods were utilized. Due to the small sample size (*n* = 15), multivariable analysis to adjust for potential confounders could not be performed; thus, all statistical associations are unadjusted. Additionally, a comprehensive correlation matrix of all analyzed variables is presented (Supplementary Table S1). First, the unadjusted correlations between immune cell density and growth metrics were assessed. This analysis showed a moderate inverse correlation between TAM density and RGR (Spearman’s *R* = −0.329, 95% CI: −0.720 to 0.221, *p* = 0.232) and a moderate positive correlation between B7-H3-positive tumor cell density and RGR (Spearman’s *R* = 0.425, 95% CI: −0.112 to 0.770, *p* = 0.114). Although these correlations did not reach statistical significance, likely due to the limited sample size (*n* = 15), the effect sizes (*R* ≈ 0.35) suggest a potential biological link between the immune microenvironment and tumor growth kinetics. No significant correlations were identified between any of the immune components and the absolute growth metric, MVC.

**Figure 4:**
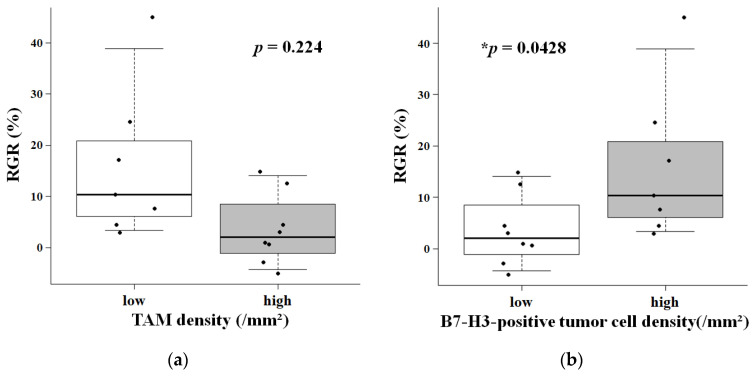
Boxplots comparing RGR between patient groups (*n* = 15) stratified by the median density of each biomarker. (**a**) The RGR showed a trend towards higher values in the low TAM density group than in the high-density group. (**b**) The RGR showed a nominally significant difference, with higher values in the high B7-H3-positive tumor cell density group than in the low-density group. *, *p* < 0.05.

To translate these biological observations into a clinically relevant context, we stratified the patients into “low” and “high” density groups based on the median value for each cell density (TAM density: 2053.3 cells/mm^2^; B7-H3-positive tumor cell density: 1828.4 cells/mm^2^). Exploratory comparative analyses between these groups suggested distinct growth trends. These median-split analyses are presented as supportive/exploratory to complement the continuous analyses described above. Tumors with low TAM density exhibited a trend towards higher RGR compared to those with high TAM density, although this difference was not statistically significant (Hodges-Lehmann estimate [Low minus High]: 5.90%, 95% CI: −5.00 to 20.20; *p* = 0.224; Figure 4a). Patients with a high density of B7-H3-positive tumor cells showed a nominally significant difference in median RGR (Hodges-Lehmann estimate [Low minus High]: −9.57%, 95% CI: −24.00 to −0.10; *p* = 0.0428, unadjusted and without multiple-testing correction; Figure 4b). Multiple testing correction was not applied in this exploratory analysis; therefore, the nominal *p*-values should be interpreted with caution due to the risk of Type I error. Individual data points are overlaid on the boxplots to demonstrate the distribution relative to the median threshold.

We performed a sensitivity analysis for the heterogeneous imaging intervals (33–1815 days). First, the relationship between imaging interval and RGR was evaluated. Spearman’s rank correlation analysis showed no significant correlation (*R* = −0.375, 95% CI: −0.745 to 0.170, *p* = 0.168), indicating that the length of the imaging interval did not systematically bias the RGR calculations. Second, to rule out the influence of long imaging intervals on growth rate estimation, we re-analyzed the data using a sub-cohort of cases with intervals of <1 year (*n* = 13). Specifically, the two cases excluded from this sub-analysis (Cases 5 and 6) had observation intervals of 1241 and 1815 days, respectively. Their RGRs (4.4% and 7.6%) fell within the cohort range (median 4.5%; range −5.1% to 45.1%). In this sensitivity analysis, the correlation trends remained consistent with the primary analysis: TAM density showed Spearman’s *R* of −0.30 (vs. −0.329 in the full cohort), and B7-H3-positive tumor cell density showed Spearman’s *R* of 0.37 (vs. 0.425 in the full cohort). The stability of these coefficients suggests that the observed trends are consistent and likely not driven solely by outliers with extended imaging intervals.

## 4. Discussion

The central finding of this study is the strong inverse correlation between TAM and B7-H3-positive tumor cell densities within the TIME (*R* = −0.921; Figure 3), suggesting that WHO grade II meningiomas exhibit an inverse continuum-like relationship. Secondarily, our exploratory analysis indicated that these macrophage-rich/B7-H3-low and macrophage-poor/B7-H3-high patterns may differ in preoperative growth behavior. Although continuous variables revealed moderate correlation coefficients (*R* ≈ 0.35) between immune components and unadjusted RGR that did not reach statistical significance, likely due to the limited statistical power of our small cohort, their direction and magnitude suggest a potential biological signal.

All reported associations between immune component densities and RGR are unadjusted for covariates—including tumor location, Simpson Grade, and adjuvant radiation—because the sample size (*n* = 15) precluded multivariable modeling. Although Simpson Grade showed a nominal correlation with MVC (*R* = 0.563, *p* = 0.029), this was not adjusted for multiple testing, and the small sample size precludes meaningful interpretation. Accordingly, residual confounding cannot be excluded, and all effect estimates should be interpreted as unadjusted exploratory associations. Building on the continuous effect sizes, we applied median-split stratification as a supplementary clinical framework. In the absence of pre-established cut-off values for meningiomas, this approach was selected to minimize selection bias and ensure reproducibility. Using this stratification, the median-split comparison raises the possibility that tumors with a macrophage-poor/B7-H3-high pattern may show faster preoperative growth: the association showed a nominally significant difference for high B7-H3 expression (*p* = 0.0428, unadjusted and without multiple-testing correction; Figure 4b), while the association with TAM density showed a non-significant trend (*p* = 0.224; Figure 4a). LOOCV (Section 3.3) confirmed the robustness of the inverse correlation, whereas the link to growth kinetics was assessed by post hoc power analysis. Formal multiple testing corrections were not applied to prevent Type II errors in hypothesis generation; however, if a Bonferroni correction for two biomarkers were applied (α = 0.025), the *p* = 0.0428 finding would not meet strict statistical significance. We therefore acknowledge the increased risk of Type I error, and this nominal finding must be interpreted as hypothesis-generating rather than a definitive conclusion. Overall, these observations raise the hypothesis that the macrophage-poor/B7-H3-high pattern identifies a subset of grade II meningiomas with faster preoperative growth; however, our data do not establish this pattern as a validated biomarker, and whether it carries prognostic or predictive value must be determined in larger, prospectively assembled cohorts.

Our findings regarding the relationship between immune patterns and tumor growth rates align with the recently reported molecular classification of WHO grade II meningiomas [5]. These genomic studies have identified four groups (MG1–MG4), which include an “immunogenic” subtype (MG1) and an aggressive “proliferative” subtype (MG4). Our “high TAM” group exhibited slower growth (Figure 4a), which may be conceptually consistent with the “immunogenic” MG1 subtype characterized by dense immune infiltration and a favorable outcome [5]. In contrast, our “high B7-H3-positive tumor cell” group exhibited rapid growth (Figure 4b), arguably similar to the “proliferative” MG4 subtype, which typically has low immune infiltration. However, these comparisons should be regarded as speculative because molecular subtyping was not performed in our cohort. These molecular studies defined the broad immune landscape but did not evaluate B7-H3 as a growth-kinetics marker; although B7-H3 expression has been reported across all meningioma grades [23,24], our exploratory data suggest that higher expression may accompany faster preoperative growth in a subset of grade II tumors. While our cross-sectional data cannot establish causality, these findings are consistent with reports in other malignancies, wherein B7-H3 promotes tumor progression by enhancing migration and invasion via tumor-intrinsic pathways [25,26,27]. Notably, in meningiomas, elevated B7-H3 expression has been associated with oncogenic mutations in the PI3K/AKT/mTOR pathway [23], which is a key driver of cell proliferation. B7-H3 also promotes tumor growth via ERK and STAT3 signaling, which may explain a threshold effect whereby high expression accelerates tumor kinetics [12,28]. Therefore, the elevated density of B7-H3-positive tumor cells in a macrophage-poor environment may reflect a tumor-intrinsic proliferative phenotype, although it remains to be determined whether B7-H3 functions as a driver or is upregulated as a consequence of rapid proliferation.

The strong inverse correlation observed in this study (*R* = −0.921; Figure 3) suggests that WHO grade II meningiomas may segregate into distinct biological profiles. This contrasts with other solid malignancies, where B7-H3 expression often correlates positively with TAM density via STAT3-dependent CCL2–CCR2-driven M2 polarization [28,29]. The divergence observed in our cohort, specifically the macrophage-poor/B7-H3-high phenotype, implies that the biological function of B7-H3 in these meningiomas may differ from the classical immunosuppressive paradigm. In the absence of TAM infiltration, the higher RGR observed in cases with a macrophage-poor/B7-H3-high pattern raises the possibility of non-immunological, tumor-intrinsic mechanisms [30,31]. Regarding the causality of this association, we must consider bidirectional possibilities. While B7-H3 may promote proliferation, it is equally plausible that rapidly proliferating tumors, which often outgrow their blood supply, leading to hypoxia, may upregulate B7-H3 expression as an adaptive response to microenvironmental stress [32]. Furthermore, the inverse TAM–B7-H3 correlation may also reflect regional heterogeneity (hypoxia, cytokine gradients) or distinct evolutionary trajectories [33], rather than direct interaction.

The key methodological observation is the utility of RGR over MVC as a biologically relevant endpoint (Table 2). RGR is the standard for meningioma volumetry and permits comparison with prior pivotal studies [14,16]; comparison with alternative models was not feasible at *n* = 15. Importantly, our sensitivity analysis restricting the cohort to intervals of <1 year (*n* = 13) demonstrated that the correlation coefficients for immune biomarkers remained stable (TAMs: *R* = −0.30; B7-H3: *R* = 0.37), mitigating concerns that calculating rates from heterogeneous intervals might introduce systematic bias. These findings suggest that the immunophenotype is associated with RGR rather than MVC in this exploratory cohort. This implies a deeper biological significance: TIME likely modulates the tumor’s intrinsic proliferative potential (a rate), which is captured by RGR, rather than the absolute volume increase (an amount), which is captured by MVC and confounded by initial tumor size [14,16,34]. Thus, RGR may provide a more accurate reflection of the biological aggressiveness associated with the immune microenvironment.

This study has several limitations. The primary constraint is the small sample size (*n* = 15), which restricts statistical power (post hoc power of 34.9% for the B7-H3-RGR correlation and 21.9% for the TAM-RGR correlation) and generalizability; this cohort size is inherently tied to the requirement for serial preoperative MRI during a “watch-and-wait” period, a clinically rare scenario for progressive WHO grade II meningiomas typically managed with immediate resection. Consequently, multivariable analysis was not feasible; all reported associations are unadjusted for confounders such as tumor location or Simpson Grade, and the wide 95% confidence intervals (e.g., −0.720 to 0.221 for the TAM-RGR linear correlation) underscore that findings must be interpreted as hypothesis-generating observations. Methodologically, the heterogeneous imaging intervals (33–1815 days) and median-split stratification introduce additional variability and preclude definitive threshold estimation. Volumetric measurements were performed by a single observer (EI) with high intra-rater reproducibility (ICC > 0.95; mean absolute percentage difference 2.57%), but inter-rater reliability was not assessed. Regarding biological characterization, the pan-macrophage marker Iba1 precludes M1/M2 differentiation [35], preventing evaluation of TAM polarization—a crucial distinction, as M2 functional heterogeneity may significantly influence tumor growth dynamics. Our immunohistochemistry (IHC) panel omitted CD8-positive T cells and NK cells, restricting comprehensive immune profiling. The QuPath classifier provided semi-automated macroscopic density approximations without independent validation or formal performance metrics, and standard single-stain IHC precluded precise microscopic spatial co-localization analysis. Finally, the cohort was not molecularly subtyped, rendering the proposed link to the MG1/MG4 molecular classification [5] strictly hypothesis-generating.

Future studies should validate these preliminary patterns in larger, multi-institutional cohorts. Given that B7-H3 overexpression is prevalent regardless of histological grade [24], future studies should also investigate whether the correlation between B7-H3 and growth kinetics transcends the WHO grading system. In addition, incorporating comprehensive molecular profiling, such as DNA methylation-based classification, will be essential to confirm whether the macrophage-poor/B7-H3-high phenotype corresponds strictly to the MG4 molecular subtype [5]. Moreover, whether this B7-H3-associated phenotype represents a viable therapeutic vulnerability remains unknown. Evaluating the efficacy of B7-H3-targeting therapies (e.g., antibody-drug conjugates or CAR-T cells) using patient-derived preclinical models of this macrophage-poor/B7-H3-high subset represents a high-priority direction for future translational research [11,12,30]. To overcome the structural limitations of standard immunohistochemistry, future studies must incorporate high-plex profiling technologies. Specifically, the application of multiplex immunohistochemistry (mIHC), highly multiplexed single-cell imaging (e.g., CODEX), or transcriptomics-based tissue profiling (e.g., Visium) will be essential [36]. By mapping the TIME at single-cell resolution, we can elucidate the mechanistic basis of the inverse TAM-B7-H3 relationship, ultimately clarifying whether this reflects distinct evolutionary trajectories driven by upstream oncogenes or true functional immune exclusion operating locally.

## 5. Conclusions

This pilot study hypothesizes an inverse relationship between TAMs and B7-H3 in WHO grade II meningiomas. Our data suggest that a macrophage-poor/B7-H3-high pattern may mark a subset of tumors with faster preoperative growth in exploratory analyses. Importantly, these findings are derived from a small, preselected cohort of tumors managed with an initial “watch-and-wait” strategy, which may represent a clinically distinct subset of WHO grade II meningiomas whose broader generalizability remains to be established. Concurrent evaluation of these TIME components may provide a valuable framework for future risk stratification. Whether B7-H3 represents a functional driver or therapeutic target in this specific subset remains a critical question for future preclinical and large-scale clinical studies.

## Figures and Tables

**Table 1:** Patient demographics and clinical characteristics.

Variable	Value or *n* (%)
Total patients	15
Age (years), median (range)	74.0 (33 to 91)
Sex, *n* (%)	
Female	7 (46.7%)
Male	8 (53.3%)
Tumor location, *n* (%)	
Non-skull base	11 (73.3%)
Skull base	4 (26.7%)
Preoperative MRI findings	
Peritumoral edema	13 (86.7%)
DWI high signal	9 (60.0%)
Interval between initial and follow-up MRIs (days), median (range)	63.0 (33 to 1815)
RGR (%/month), median (range)	4.5 (−5.1 to 45.1)
MVC (cm^3^/month), median (range)	1.51 (−3.36 to 16.68)
Extent of resection (Simpson Grade)	
I–II	7 (46.7%)
III–IV	8 (53.3%)
V	0 (0%)
Postoperative radiation therapy	8 (53.3%)
Postoperative follow-up duration (months), median (range)	36 (11 to 136)
Recurrence	0 (0%)

DWI, diffusion-weighted imaging; MRI, magnetic resonance imaging; MVC, monthly volume change; RGR, relative growth rate.

**Table 2:** Association Between TIME Indicators and Growth Kinetics (RGR/MVC) in WHO Grade II Meningiomas.

Case No.	Age	Sex	RGR (%/Month)	MVC (cm^3^/Month)	Tumor Cell Density (Cells/mm^2^)	CD4-Positive T Cell Density (Cells/mm^2^)	TAM Density (Cells/mm^2^)	B7-H3-Positive Tumor Cell Density (Cells/mm^2^)
1	47	M	2.9	1.00	3521.327	1.896	667.962	3475.581
2	79	F	45.1	16.68	3412.426	0.543	986.211	3141.020
3	71	M	−2.9	−0.42	1277.836	0.373	2820.585	1198.145
4	74	M	0.9	0.63	1745.805	0.563	2071.023	1552.060
5	58	M	4.4	3.98	1871.724	1.298	2635.204	1828.401
6	79	F	7.6	1.51	2028.721	3.104	1421.038	1988.756
7	91	F	10.4	3.43	4856.738	0.087	167.291	4761.318
8	76	M	4.5	0.96	4633.769	0.050	704.694	4387.715
9	51	F	0.6	0.17	1634.831	0.004	3267.911	1539.134
10	74	M	−5.1	−3.36	1939.904	0.381	2053.308	1810.619
11	49	F	24.6	4.47	3511.764	0.176	890.771	3432.311
12	33	F	12.6	2.01	1766.002	0.574	2512.692	1697.196
13	34	F	3.1	0.27	1609.494	0.283	2403.925	1553.730
14	84	M	17.2	6.86	3494.157	0.343	1601.837	3329.979
15	84	M	14.8	5.23	1349.496	1.835	3107.742	1251.115

F, female; M, male; MVC, monthly volume change; No., patient number; RGR, relative growth rate.

## Data Availability

The datasets generated and analyzed during the current study are not publicly available because of institutional policy and the risk of compromising patient privacy in this small cohort. De-identified data may be made available from the corresponding author upon reasonable request and with permission from the Institutional Review Board of Aichi Medical University.

## Supplementary Materials

The following supporting information can be downloaded at https://www.mdpi.com/article/10.3390/cancers18101545/s1, Table S1: Spearman’s rank correlation matrix of all analyzed clinical and immunological variables.

## Abbreviations

The following abbreviations are used in this manuscript:

DAB	Diaminobenzidine
DWI	Diffusion-weighted imaging
IHC	Immunohistochemistry
mAbs	Monoclonal antibodies
MRI	Magnetic resonance imaging
MVC	Monthly volume change
RGR	Relative growth rate
TAM	Tumor-associated macrophage
TIME	Tumor immune microenvironment
WHO	World Health Organization
