# Supplementary material for: Association of Iba1-Positive Macrophages and B7-H3-Positive Tumor Cells with Tumor Growth Kinetics in WHO Grade II Meningioma: A Pilot Watch-and-Wait Cohort Study
Source: Cancers (Basel). 2026 May 10;18(10):1545. doi: 10.3390/cancers18101545 (PMC13204069; doi:10.3390/cancers18101545)
Supplement: Supplementary file 1 [file cancers-18-01545-s001.zip › cancers-4247237-supplementary.pdf]

Supplementary Table S1. Spearman's rank correlation matrix of all analyzed clinical and immunological variables.

Variables	TAM	B7-H3	CD4	RGR	MVC	Volume	Interval	Simpson
**TAM density**								
**B7-H3-positive tumor cell density**	**-0.921** *(<0.001)****							
**CD4-positive T cell density**	0.096 *(0.732)*	-0.168 *(0.550)*						
**RGR**	-0.329 *(0.232)*	0.425 *(0.114)*	0.057 *(0.840)*					
**MVC**	-0.257 *(0.355)*	0.396 *(0.143)*	0.221 *(0.428)*	**0.929** *(<0.001)****				
**Initial Tumor Volume**	-0.146 *(0.603)*	0.164 *(0.558)*	0.043 *(0.879)*	-0.196 *(0.483)*	0.050 *(0.860)*			
**Interval between initial and follow-up MRIs**	0.475 *(0.073)*	-0.438 *(0.103)*	0.327 *(0.234)*	-0.375 *(0.168)*	-0.298 *(0.280)*	-0.349 *(0.203)*		
**Simpson Grade**	-0.361 *(0.187)*	0.481 *(0.069)*	0.267 *(0.335)*	0.380 *(0.162)*	**0.563** *(0.029)**	0.252 *(0.366)*	0.171 *(0.543)*	

Data are presented as Spearman's rank correlation coefficient (*R*) with the corresponding unadjusted *p*-value in parentheses. Statistically significant correlations are highlighted in bold (*, $p < 0.05$; ***, $p < 0.001$). TAM, tumor-associated macrophage; RGR, relative growth rate; MVC, monthly volume change.
